# Virulence Determinants and Multidrug Resistance of *Escherichia coli* Isolated from Migratory Birds

**DOI:** 10.3390/antibiotics10020190

**Published:** 2021-02-15

**Authors:** Md. Saiful Islam, Md. Mehedi Hasan Nayeem, Md. Abdus Sobur, Samina Ievy, Md. Amirul Islam, Saifur Rahman, Md. Abdul Kafi, Hossam M. Ashour, Md. Tanvir Rahman

**Affiliations:** 1 Department of Microbiology and Hygiene, Faculty of Veterinary Science, Bangladesh Agricultural University, Mymensingh 2202, Bangladesh; dvm41257@bau.edu.bd (M.S.I.); mhnayeem386@gmail.com (M.M.H.N.); soburvetbau@gmail.com (M.A.S.); v.samina@gmail.com (S.I.); amirulmicrobau@gmail.com (M.A.I.); saifurrahman@bau.edu.bd (S.R.); makafi2003@bau.edu.bd (M.A.K.); 2 Department of Integrative Biology, College of Arts and Sciences, University of South Florida, St. Petersburg, FL 33701, USA; 3 Department of Microbiology and Immunology, Faculty of Pharmacy, Cairo University, Cairo 11562, Egypt

**Keywords:** migratory birds, *E. coli*, virulence, APEC, MDR, environment, public health

## Abstract

Migratory birds are carriers of multidrug resistant pathogenic *Escherichia coli*. However, their roles in the dissemination of these resistant pathogens are still being neglected in Bangladesh. The present study was therefore carried out to detect multidrug resistant *E. coli*. In addition, these isolates were also screened for the presence of avian pathogenic *E. coli* (APEC)-associated virulence genes. A total of 66 fecal matter samples of migratory birds were screened. *E. coli* were isolated and identified by culturing and biochemical tests followed by polymerase chain reaction (PCR). APEC-associated virulence genes were detected by PCR. Disk diffusion assays were employed to investigate antibiogram profiles. Bivariate analysis was performed to assess correlations in resistance patterns between antimicrobials and to assess associations between virulence genes of *E. coli*. Among the 66 samples assessed by PCR, 55 (83.33%) were found positive for *E. coli.* Of these 55 isolates, the APEC-associated virulence gene *fimC* was detected in 67.27% of the isolates, which was significantly higher than in the cases of *iucD* (29.09%) and *papC* (5.45%) genes. In addition, three isolates were found positive for all three virulence genes, while 23 and 12 isolates were positive for one and two virulence genes respectively. In the bivariate analysis, significant associations were detected between *fimC* and *iucD* virulence genes. Using the antibiogram, all *E. coli* isolates were found to be multidrug resistant (MDR). The isolates exhibited 100% resistance against ampicillin and erythromycin in addition to varying percentages of resistance against streptomycin, tetracycline, ciprofloxacin, and chloramphenicol. Highly positive correlations between tetracycline and ciprofloxacin, chloramphenicol and ciprofloxacin, chloramphenicol and tetracycline were observed by bivariate analysis. To the best of our knowledge, this is the first study that reports APEC-associated virulence genes of MDR *E. coli* from migratory birds in Bangladesh. Results indicate that migratory birds are reservoirs of MDR *E. coli* isolates carrying APEC-associated virulence genes, which can seriously contribute to the development of human and animal diseases.

## 1. Introduction

There are over 10,000 known species of birds that are distributed globally [1]. Birds can travel long distances between countries and across continents. Bangladesh is located in the subtropical region and thus has milder winters than in the northern hemisphere. During the winter season, migratory birds travel to Bangladesh, and inhabit suitable water bodies such as ponds, lakes, and rivers. Migratory birds are known to be involved in the transmission and spread of human and animal pathogens such as bacteria, viruses, fungi, archaea, and parasites as healthy carriers or as hosts of infected vectors [2,3]. Several studies revealed the transmission patterns of bacterial pathogens to aquatic environment from migratory birds [4,5]. Importantly, bacterial pathogens can be transmitted to humans, animals, and poultry by ducks and duck-like birds from water bodies contaminated by fecal matter of migratory birds. In addition, people dwelling around water bodies where migratory birds take rest, may come in contact with contaminated water that they may try to use for household or agricultural purposes. Furthermore, when people usually use contaminated water for dairy or poultry farming, bacterial pathogens can be transmitted to other humans and animals.

Among the different bacteria transmitted by migratory birds, *Escherichia coli* are important commensal avian and human pathogens that exist as part of the microbiota of the intestinal tract of avian species [6]. Pathogenic *E. coli* can infect the respiratory tract, urinary tract, and bloodstream of humans and animals [7]. Strikingly, more than 80% of urinary tract infections in humans are caused by this microorganism [8].

Avian pathogenic *E. coli* (APEC) causes avian colibacillosis in poultry, which is an infectious disease that negatively impacts the poultry sector [9]. It causes yolk sac infections, pericarditis, synovitis, peritonitis, osteomyelitis, and salpingitis in poultry [10]. The disease has been associated with several virulence genes, including *fimC*, *fimH*, *papC*, *iss*, *stx1*, *stx2*, *tss, cvi*, and *iucD* [10,11,12,13]. Virulence factors include invasins, adhesins, protectins, iron acquisition systems, and toxins and are crucial for invasion, colonization, and adherence of the pathogen to the surface of respiratory tract, its resistance mechanisms, its ability to multiply under iron-restricted situation, and its cytotoxic effects [10,14]. Among virulence genes, *fimC* (Type 1 fimbriae C) is responsible for adherence and colonization on epithelial cells, *iucD* (iron-uptake systems of *E. coli* D) is responsible for iron-acquisition, and *papC* (pyelonephritis-associated pili C) is responsible for bacterial adhesion [10]. The number and combination of virulence genes associated with APEC determine its overall virulence [15].

APEC infections are zoonotic in nature and have phylogenic similarities with uropathogenic *E. coli* (UPEC) that causes urinary tract infections and with neonatal meningitis *E. coli* that causes neonatal meningitis in humans [16,17]. In addition, these strains can share virulence factors by transmitting virulence genes and plasmids [16]. Furthermore, APEC in meat of healthy birds can be transmitted to humans via the food chain leading to extraintestinal diseases and other diseases [18].

Antimicrobial resistance (AMR) is a serious global problem that jeopardizes human, animal, and environmental health. If not contained by 2050, AMR is estimated to cause hundreds of millions of human deaths, severe financial losses, and a significant fall in livestock production [19]. The impact will be severe in low- and middle-income countries (LMICs) in Africa and Asia including Bangladesh. Migratory birds can spread antibiotic resistant pathogens over long distances to remote locations and can act as reservoirs of antibiotic-resistant bacteria [20,21,22]. They have been recognized as an important source for the environmental dissemination of AMR [21,23,24]. Multidrug resistant (MDR) *E. coli* has been from migratory birds in different parts of the world [25,26,27,28]. Fecal transmission of MDR *E. coli* from migratory birds to water bodies in different areas has been reported [29,30].

AMR has been extensively studied in humans, livestock, and poultry. There has been less focus on AMR in non-typical hosts such as in migratory birds and significant gaps of knowledge do exist. MDR *E. coli* has been reported in migratory birds in Bangladesh [25]. However, to the best of our knowledge, there is no data on virulence determinants of MDR *E. coli* associated with the APEC pathotype in Bangladesh. In this study, we assessed the hypothesis that APEC-associated virulence determinants exist in the microbiota of migratory birds travelling to Bangladesh. In addition to the isolation and identification of the virulence determinants, we assessed multidrug resistance in these migratory birds.

## 2. Results

### 2.1. Prevalence of E. coli Isolates

Out of 66 samples, the characteristic colonies of *E. coli* were observed in 62 samples (93.94%). All 62 isolates were also confirmed to be *E. coli* by Gram staining and biochemical tests. PCR results revealed that 55 of the 62 isolates (83.33%) were positive for the *malB* gene (585 bp).

### 2.2. Prevalence of the Virulence Genes Associated with the APEC Pathotype

Of the 55 *E. coli* isolates, 37 (67.27%), 16 (29.09%), and three (5.45%) were positive for *fimC*, *iucD*, and *papC* respectively (Table 1). Overall, 38/55 (69.09%) *E. coli* isolates were positive for at least one of the three APEC-associated virulence genes. Among the 38 *E. coli* isolates harboring APEC-associated virulence genes, three isolates were positive for all three virulence genes (*fimC*, *iucD*, and *papC*); 12 were positive for two virulence genes (*fimC* and *iucD*), and 23 were positive for a single virulence gene (22 for *fimC* and one for *iucD*). Based on statistical analysis, the prevalence of *fimC* was significantly higher than the other two virulence genes (chi-square test, 95% CI, *p* < 0.001) (Table 1).

### 2.3. Pearson Correlation Coefficients for Pairs of APEC-Associated Virulence Genes

A bivariate analysis conducted on APEC-associated virulence genes showed a significant correlation between *fimC* and *iucD* (Pearson correlation coefficient, *ρ* = 0.447; *p* = 0.001). There were weaker correlations between *fimC* and *papC* (Pearson correlation coefficient, *ρ* = 0.168; *p* > 0.05) and between *iucD* and *papC* (*ρ* = 0.199; *p* > 0.05) (Table 2).

### 2.4. Antibiogram Profiles of Isolated E. coli Associated with the APEC Pathotype

**Figure 1:**
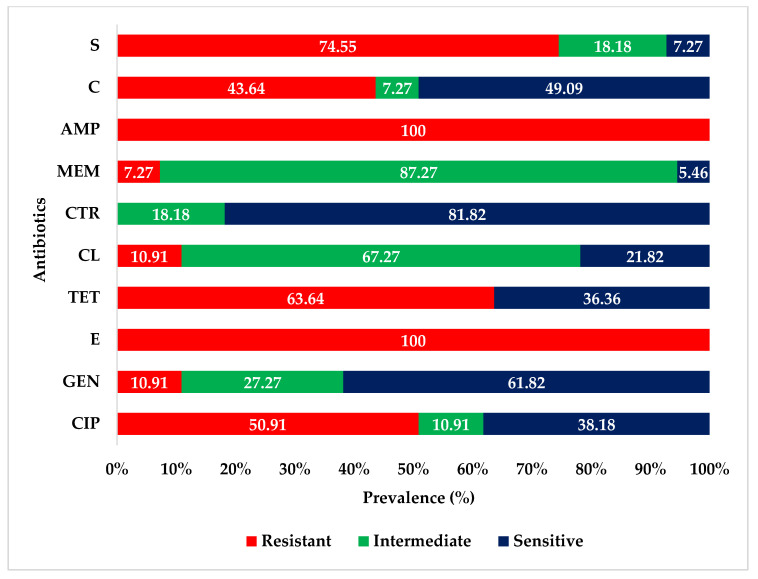
Antibiogram profiles of *E. coli* isolated from fecal samples of migratory birds. CIP = ciprofloxacin; GEN = gentamicin; E = erythromycin; TE = tetracycline; CL = colistin; CTR = ceftriaxone; MEM = meropenem; AMP = ampicillin; C = chloramphenicol; S = streptomycin.

Antibiotic sensitivity test revealed that all 55 *E. coli* isolates were highly resistant to ampicillin and erythromycin. The isolates exhibited varying degrees of resistance to streptomycin (74.55%), tetracycline (63.64%), ciprofloxacin (50.91%), and chloramphenicol (43.64%). The isolates were highly or intermediately sensitive to ceftriaxone, meropenem, gentamicin, and colistin. The antibiogram profiles of the isolated *E. coli* are presented in Figure 1.

### 2.5. Pearson Correlation Coefficients for Pairs of Antibiotics to Assess Antibiotic-Resistant E. coli Isolates

Pearson correlation tests were used to assess correlations between different antibiotic-resistant *E. coli* isolates. There were positive significant correlations between resistance patterns against tetracycline and ciprofloxacin (*ρ* = 0.694; *p* < 0.001); chloramphenicol and ciprofloxacin (*ρ* = 0.717; *p* < 0.001); chloramphenicol and tetracycline (*ρ* = 0.589; *p* < 0.001); and streptomycin and tetracycline (*ρ* = 0.426; *p* = 0.001). On the other hand, there were negative significant correlations between resistance patterns against colistin and ciprofloxacin (*ρ* = −0.356; *p* = 0.008); colistin and tetracycline (*ρ* = −0.342; *p* = 0.011); meropenem and ciprofloxacin (*ρ* = −0.285; *p* = 0.035); chloramphenicol and colistin (*ρ* = −0.308; *p* = 0.022); and streptomycin and colistin (*ρ* = −0.331; *p* = 0.014) (Table 3).

### 2.6. Prevalence of MDR E. coli

All the *E. coli* isolates (*n* = 55) were MDR in nature. Overall, 17 antibiotic resistance patterns were observed among the isolated *E. coli*. Of them, resistance pattern no. 16 (CIP, E, TE, AMP, C, S) was the most prevalent (29.09%), followed by pattern no. 3 (E, AMP, S) in 16.36% of the *E. coli* isolates. Two isolates exhibited resistance against seven antibiotics representing six classes (pattern no. 17) (Table 4).

## 3. Discussion

Migratory birds contribute to the circulation and dissemination of different bacteria including *E. coli*. As an enteric microorganism, pathogenic *E. coli* can cause both human and animal diseases and is known to develop antimicrobial resistance [31]. Since migratory birds can spread antibiotic-resistant *E. coli* during migration, we investigated fecal matter of migratory birds to identify APEC-associated virulence genes.

The 83.33% (55/66) prevalence rate of *E. coli* in migratory birds was close to the prevalence rate of a study in Portugal (85.7%) [23], but higher than prevalence rates reported in other studies in Bangladesh [25,32], Czech Republic [33], Egypt [34], Northern Italy (33.9%) [35], and Italy (24.31%) [36]. On the other hand, the prevalence rate was lower than the prevalence rate reported in a study in Saudi Arabia (94%) [26]. These variations can be due to the variations in detection methods, geographical and seasonal distribution, sample sizes, and types and species of migratory birds. In addition, stressful conditions that migratory birds experience during migration may impact the shedding rate of bacteria [37]. The detection of *E. coli* in the fecal samples of the migratory birds is not unusual because of commensal nature of this organism in the intestines of humans, animals, and avian species. In addition, migratory birds usually occupy variegated ecological niches and adapt varying feeding patterns during their different forms of migration. During these migrations, birds can host *E. coli* and contribute to its transmission from one place to another.

This is the first study to detect virulence determinants of MDR *E. coli* associated with the APEC pathotype from migratory birds in Bangladesh. Virulence genes are pivotal for the detection of any pathogenic microorganisms [38]. In the present study, 69.09% (38/55) *E. coli* isolates were positive for at least one or more virulence genes. Among them, the three tested virulence genes *(fimC, iucD*, and *papC)* were present together in only three isolates. However, the prevalence of the *fimC* (67.27%) gene was higher than *iucD* (29.09%) and *papC* (5.45%) genes. In addition, there was a significant correlation between *fimC* and *iucD* genes; but none with the *papC* gene. Previous studies reported the detection of APEC-associated virulence genes from migratory birds in Italy and Slovakia [39,40]. Both *fimC* (a chaperone-like periplasmic protein) and *papC* allow *E. coli* to adhere to host cells [41]. The *iucD* gene contributes to APEC pathogenesis through an iron-acquisition system and the mediation of aerobactin synthesis [42]. As APEC colonizes its avian hosts, it can spread through the fecal route to the environment potentially threatening humans, animals, and other avian hosts [14]. Furthermore, biological and environmental stress factors can compound the APEC’s threat to the livestock and human population [43].

It is noteworthy that APEC-associated virulence genes isolated from migratory birds in this study have been previously detected in humans and wild mammals [44]. Thus, migratory birds can act as potential reservoirs for antibiotic-resistant APEC which, due to its zoonotic nature, can spread from these birds to the human population [17,22,45]. In line with earlier studies [45,46], we detected multidrug resistance in the isolated *E. coli*. All the *E. coli* isolates exhibited very high resistance against ampicillin and erythromycin, in addition to different levels of resistance against streptomycin, tetracycline, ciprofloxacin, and chloramphenicol. Previous studies reported resistance in *E. coli* isolated from migratory birds against ampicillin, ciprofloxacin, chloramphenicol, tetracycline, streptomycin, gentamicin [26,28,33,36,47]. Notably, colistin-resistant and meropenem-resistant *E. coli* have also been detected in this study. Colistin is in the reserve group of antibiotics and its detection in isolated *E. coli* is alarming. In addition, meropenem is from the carbapenem group which is typically used for the treatment of serious infections in humans only. Migratory birds might have obtained these resistant isolates from an environment contaminated with human secretions or excretions. Further studies at the molecular level need to be conducted to follow up on this finding.

In this study, we reported significant positive correlations (*p* < 0.05) between the resistance profiles of ciprofloxacin and tetracycline, chloramphenicol and ciprofloxacin, chloramphenicol and tetracycline, and streptomycin and tetracycline; significant inverse correlations were observed in between colistin and ciprofloxacin, colistin and tetracycline, meropenem and ciprofloxacin, chloramphenicol and colistin, and streptomycin and colistin. The significant correlations observed between antimicrobials might be related to the haphazard use of antibiotics in animals and poultry in areas inhabited by the migratory birds. Cross-contamination of the environment, such as the water bodies, might also have played a role.

It is worth mentioning that all the *E. coli* isolates from migratory birds in this study were MDR in nature. The detection of MDR *E. coli* from the migratory birds is not uncommon. Previously, Hasan et al. [25] reported MDR *E. coli* in fecal samples of migratory birds in Bangladesh. Similar observations have also been made in other parts of the world [26,28]. Our findings leave open the possibility of long-distance transmissions of MDR bacteria from their original habitats to far locations, which can be very alarming, especially if the transmission was to areas in which the public has not been educated about infectious disease control and prevention [48]. The transmission of resistant *E. coli* from livestock to wild birds has previously been reported [49]. It is likely to occur in Bangladesh as people tend to keep their animals and poultry near water bodies where interaction with migratory birds is possible. Antimicrobial resistant *E. coli* has also been detected in wild mammals [50,51].

Aquatic environments are considered to be hotspots for the transmission of antibiotic-resistant bacteria such as *E. coli* [52]. Along this line, previous studies reported that ducks, which live near water bodies, can transmit antibiotic-resistant bacteria [53,54]. Since migratory birds carrying antibiotic-resistant *E. coli* inhabit water bodies, they might be contributing to the dissemination of antibiotic-resistant *E. coli* through fecal matter to the surrounding aquatic environments, which can jeopardize human and animal health directly or indirectly. One major area of concern is if the resistant bacteria gain entry into the human food chain. Given all the above, it will be important to control and prevent the spread of antibiotic-resistant bacteria from migratory birds to humans, animals, and other poultry.

## 4. Materials and Methods

### 4.1. Study Area

**Figure 2:**
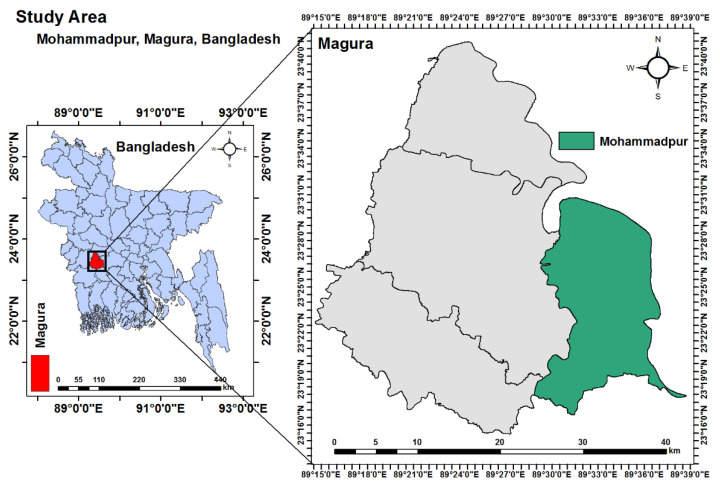
Study area map, created with ArcMap 10.7 software (ESRI, Redlands, CA, USA) based on Geographical Information System (GIS).

The present study was carried out in Baojani Baor within the Mohammadpur Upazila (23.4056° N, 89.5686° E) of the Magura district of Bangladesh (Figure 2) during the period of November 2019 to November 2020. The area was selected due to the abundance of wintering migratory birds in this area every year. Humans in the area typically work in agriculture or animal rearing. In addition, different poultry species are frequently available in the area.

### 4.2. Sampling and Initial Processing

A total of 66 freshly dropped wet fecal samples from migratory birds were collected from the ground and tree leaves. Based on the procedure of Akter et al. [55], samples were collected using sterilized cotton buds and were transferred to separate sterilized zip lock bags with particular tag numbers followed by transferring to the laboratory while maintaining cold chain throughout. Each sample was added to 5 mL nutrient broth (HiMedia, India) in a sterilized test tube. All the test tubes containing fecal samples were then incubated aerobically overnight at 37 °C.

### 4.3. Isolation of E. coli

The isolation of *E. coli* was done by culturing on Eosin Methylene Blue (EMB) agar (HiMedia, India) plates. Initially, the overnight grown broth cultures were streaked on EMB agar plates with sterilized inoculating loops. Subsequently, the inoculated agar plates were incubated at 37 °C for overnight to obtain pure colonies. If needed for securing pure colonies, subcultures were conducted on EMB agar plates. The growth of single green-colored metallic sheen colonies on EMB agar plates indicated the growth of *E. coli*. The single pure colonies were screened for further confirmation by Gram’s staining technique and different biochemical tests including motility test, catalase test, coagulase test, sugar fermentation tests, methyl red test, Voges–Proskauer test, and indole tests [56,57,58].

### 4.4. Molecular Detection of E. coli

The final confirmation of *E. coli* was done by polymerase chain reaction (PCR) assays targeting the *malB* gene (Table 5).

For PCR, the genomic DNA was extracted from pure cultures of *E. coli* by the boiling method as previously described [61,62]. In brief, a pure colony from freshly grown culture was added into 100 µL phosphate buffer solution (PBS) in a sterile Eppendorf tube and mixed by gentle vortexing. Subsequently, the mixture was boiled and cooled for 10 min in each step, followed by the centrifugation at 10,000 rpm for 10 min. Finally, the supernatant was collected (genomic DNA) and stored at −20 °C for further use.

The PCR was performed with a final volume of 20 µL containing 4 µL nuclease free water, 10 µL master mix (2X) (Promega, Madison, WI, USA), 1 µL of forward and reverse primers, and 4 µL of genomic DNA. After completion, the amplified PCR products were examined by gel electrophoresis with 1.5% agarose. Finally, staining and visualization of the amplicon products were done in ethidium-bromide and under an ultraviolet trans-illuminator (Biometra, Göttingen, Germany). The targeted amplicon sizes were checked using 1 kb DNA ladder ((Promega, Madison, WI, USA).

### 4.5. Molecular Detection of APEC-Associated Virulence Genes

In order to detect the virulence determinants of *E. coli* isolates, three genes (*fimC*, *iucD*, and *papC*) associated with the APEC pathotype were selected. These genes were previously reported in APEC isolates from different poultry species [10,18,42,63]. The *E. coli* isolates (confirmed by PCR) were screened to detect virulence genes associated with the APEC pathotype from migratory birds. The presence of the virulence gene *iucD* indicates pathogenic *E. coli*. We listed the primers used in this study along with their target genes in Table 5.

### 4.6. Antibiotic Susceptibility Test

The Kirby–Bauer disk diffusion test [64] was used for antibiotic susceptibility testing of isolated *E. coli* in accordance with the guidelines of the Clinical and Laboratory Standards Institute [65]. Ten antibiotics belonging to nine antimicrobial classes were used. Here is a list of the nine classes and their associated antibiotics: fluoroquinolones (ciprofloxacin—5 µg), aminoglycosides (gentamicin—10 µg; and streptomycin—10 µg), macrolides (erythromycin—15 µg), tetracycline (tetracycline—30 µg), polypeptides (colistin—10 µg), cephalosporins (ceftriaxone—30 µg), carbapenems (meropenem—10 µg), penicillins (ampicillin—25 µg), and amphenicols (chloramphenicol—10 µg). The sensitivity tests were carried out on freshly-grown bacteria in nutrient broth using Mueller–Hinton agar (HiMedia, India) plates. The concentration of bacteria was adjusted to 0.5 McFarland (HiMedia, India) units before testing. A Multidrug resistant isolate was defined as an isolate that is resistant to three or more classes of antimicrobial agents [66].

### 4.7. Statistical Analyses

#### 4.7.1. Descriptive Analysis

Data entry was done using Microsoft Excel 2013 (Los Angeles, CA, USA) and analysis was performed using the Statistical Package for Social Science- SPSS (IBM SPSS 25, IBM, Chicago, IL, USA).Variations in the prevalence of APEC-associated virulence genes were assessed by the chi-square test for goodness-of-fit using SPSS. A *p*-value less than 0.05 was considered to be statistically significant.

#### 4.7.2. Bivariate Analysis

Bivariate analysis was performed to assess correlation in resistance patterns in pairs of antibiotics and for correlation in pairs of APEC-associated virulence genes from the isolated *E. coli*. A *p*-value less than 0.05 (*p* < 0.05) was deemed statistically significant.

## 5. Conclusions

This is the first study to detect virulence genes of MDR *E. coli* associated with the APEC pathotype isolated from migratory birds in Bangladesh. These migratory birds might spread antibiotic-resistant *E. coli* to the environment, which can impact human and animal health. Active surveillance for migratory birds is important together with the implementation of the one health approach to control the zoonotic potential of APEC and to minimize the AMR-associated health hazards [67].

## Figures and Tables

**Table 1:** Prevalence of avian pathogenic *E. coli* (APEC)-associated virulence genes in *E. coli* isolated from fecal matter of migratory birds.

Virulence Gene	Occurrence (%) (*n* = 55)	*p*-Value *
*fimC*	37 (67.27)	<0.001
*iucD*	16 (29.09)	
*papC*	3 (5.45)	

* A *p*-value less than 0.05 (*p* < 0.05) was deemed as significant.

**Table 2:** Pearson correlation coefficients for pairs of APEC-associated virulence genes isolated from fecal matter of migratory birds.

	Statistical Analysis	*fimC*	*iucD*	*papC*
*fimC*	Pearson Correlation Coefficient	1		
	*p*-value (two-tailed)	-		
*iucD*	Pearson Correlation Coefficient	0.447 ^‡^	1	
	*p*-value (two-tailed)	0.001 *	-	
*papC*	Pearson Correlation Coefficient	0.168	0.199	1
	*p*-value (two-tailed)	0.221	0.146	-

* A *p*-value less than 0.05 (*p* < 0.05) was deemed as significant; ‡ correlation is significant at the 0.01 level (two-tailed).

**Table 3:** Pearson correlation coefficients for pairs of antibiotics to assess antibiotic-resistant *E. coli* isolates from fecal samples of migratory birds.

	Statistical Analysis	CIP	GEN	E	TE	CL	CTR	MEM	AMP	C	S
CIP	Pearson Correlation Coefficient	1									
	*p*-value (two-tailed)	-									
GEN	Pearson Correlation Coefficient	0.123	1								
	*p*-value (two-tailed)	0.371	-								
E	Pearson Correlation Coefficient	- ^a^	- ^a^	- ^a^							
	*p*-value (two-tailed)	-	-	-							
TE	Pearson Correlation Coefficient	0.694 ^‡^	0.099	- ^a^	1						
	*p*-value (two-tailed)	0.000 *	0.471	-	-						
CL	Pearson Correlation Coefficient	0.356 ^‡^	0.065	- ^a^	−0.342 ^†^	1					
	*p*-value (two-tailed)	0.008 *	0.639	-	0.011 *	-					
CTR	Pearson Correlation Coefficient	- ^a^	- ^a^	- ^a^	- ^a^	- ^a^	- ^a^				
	*p*-value (two-tailed)	-	-	-	-	-	-				
MEM	Pearson Correlation Coefficient	0.285 ^†^	0.098	- ^a^	-0.225	0.098	- ^a^	1			
	*p*-value (two-tailed)	0.035 *	0.477	-	0.099	0.477	-	-			
AMP	Pearson Correlation Coefficient	- ^a^	- ^a^	- ^a^	- ^a^	- ^a^	- ^a^	- ^a^	- ^a^		
	*p*-value (two-tailed)	-	-	-	-	-	-	-	-		
C	Pearson Correlation Coefficient	0.717 ^‡^	0.073	- ^a^	0.589 ^‡^	−0.308 ^†^	- ^a^	−0.246	- ^a^	1	
	*p*-value (two-tailed)	0.000 *	0.598	-	0.000 *	0.022 *	-	0.070	-	-	
S	Pearson Correlation Coefficient	0.261	0.063	- ^a^	0.426 ^‡^	−0.331 ^†^	- ^a^	−0.158	- ^a^	0.178	1
	*p*-value (two-tailed)	0.054	0.646	-	0.001 *	0.014 *	-	0.250	-	0.195	-

* A *p*-value less than 0.05 (*p* < 0.05) was deemed as significant; ^‡^ correlation is significant at the 0.01 level (two-tailed); † correlation is significant at the 0.05 level (two-tailed); ^a^ cannot be computed because at least one of the variables is constant; CIP = ciprofloxacin; GEN = gentamicin; E = erythromycin; TE = tetracycline; CL = colistin; CTR = ceftriaxone; MEM = meropenem; AMP = ampicillin; C = chloramphenicol; S = streptomycin.

**Table 4:** Phenotypic resistance patterns of multidrug-resistant (MDR) *E. coli* isolated from fecal matter of migratory birds.

Pattern No.	Antibiotic Resistance Patterns	No. of Antibiotics (Classes)	No. of MDR Isolates (%)	Overall No. of MDR Isolates (%)
1	E, TE, AMP	3 (3)	2 (3.64)	55 (100)
2	GEN, E, AMP	3 (3)	2 (3.64)	
3	E, AMP, S	3 (3)	9 (16.36)	
4	E, CL, AMP	3 (3)	4 (7.27)	
5	E, MEM, AMP	3 (3)	2 (3.64)	
6	CIP, E, AMP, C	4 (4)	1 (1.81)	
7	E, TE, AMP, S	4 (4)	1 (1.81)	
8	E, MEM, AMP, S	4 (4)	1 (1.81)	
9	GEN, E, TE, AMP, S	5 (4)	1 (1.81)	
10	GEN, E, CL, AMP, S	5 (4)	1 (1.81)	
11	CIP, E, TE, AMP, C	5 (5)	3 (5.45)	
12	CIP, E, TE, AMP, S	5 (5)	6 (10.91)	
13	E, TE, AMP, C, S	5 (5)	2 (3.64)	
14	E, TE, CL, AMP, S	5 (5)	1 (1.81)	
15	E, TE, MEM, AMP, S	5 (5)	1 (1.81)	
16	CIP, E, TE, AMP, C, S	6 (6)	16 (29.09)	
17	CIP, GEN, E, TE, AMP, C, S	7 (6)	2 (3.64)	

CIP = ciprofloxacin; GEN = gentamicin; E = erythromycin; TE = tetracycline; CL = colistin; CTR = ceftriaxone; MEM = meropenem; AMP = ampicillin; C = chloramphenicol; S = streptomycin; MDR = multidrug resistant.

**Table 5:** Primers used in the present study.

Target Genes	Primer Sequence (5’–3’)	Amplicon Size (bp)	Annealing Temperature (°C)	References
*malB*	F:GACCTCGGTTTAGTTCACAGA R: CACACGCTGACGCTGACCA	585	55	[59]
*fimC*	F: GGTAGAAAATGCCGATGGTG R: CGTCATTTTGGGGGTAAGTGC	496	59	[60]
*iucD*	F: ACAAAAAGTTCTATCGCTTCC R: CCTGATCCAGCTGATGCTC	692	55	
*papC*	F: TGATATCACGCAGTCAGTAGC R: CCGGCCATATTCACATAA	483	59	

## Data Availability

Not applicable.
